# An *In Vitro* Culture System for Long-Term Expansion of Epithelial and Mesenchymal Salivary Gland Cells: Role of TGF-*β*1 in Salivary Gland Epithelial and Mesenchymal Differentiation

**DOI:** 10.1155/2013/815895

**Published:** 2013-06-09

**Authors:** Kajohnkiart Janebodin, Worakanya Buranaphatthana, Nicholas Ieronimakis, Aislinn L. Hays, Morayma Reyes

**Affiliations:** ^1^Department of Oral Health Sciences, School of Dentistry, University of Washington, Seattle, WA 98195, USA; ^2^Department of Anatomy, Faculty of Dentistry, Mahidol University, Bangkok 10400, Thailand; ^3^Department of Oral Biology and Diagnostic Sciences, Faculty of Dentistry, Chiang Mai University, Chiang Mai 50200, Thailand; ^4^Department of Pathology, School of Medicine, University of Washington, Seattle, WA 98195, USA; ^5^Department of Laboratory Medicine, School of Medicine, University of Washington, Seattle, WA 98195, USA

## Abstract

Despite a pivotal role in salivary gland development, homeostasis, and disease, the role of salivary gland mesenchyme is not well understood. In this study, we used the *Col1a1-GFP* mouse model to characterize the salivary gland mesenchyme *in vitro* and *in vivo*. The *Col1a1-GFP* transgene was exclusively expressed in the salivary gland mesenchyme. *Ex vivo* culture of mixed salivary gland cells in DMEM plus serum medium allowed long-term expansion of salivary gland epithelial and mesenchymal cells. The role of TGF-*β*1 in salivary gland development and disease is complex. Therefore, we used this *in vitro* culture system to study the effects of TGF-*β*1 on salivary gland cell differentiation. TGF-*β*1 induced the expression of collagen, and inhibited the formation of acini-like structures in close proximity to mesenchymal cells, which adapted a fibroblastic phenotype. In contrast, TGF-*β*R1 inhibition increased acini genes and fibroblast growth factors (*Fgf-7* and *Fgf-10*), decreased collagen and induced formation of larger, mature acini-like structures. Thus, inhibition of TGF-*β* signaling may be beneficial for salivary gland differentiation; however, due to differential effects of TGF-*β*1 in salivary gland epithelial versus mesenchymal cells, selective inhibition is desirable. In conclusion, this mixed salivary gland cell culture system can be used to study epithelial-mesenchymal interactions and the effects of differentiating inducers and inhibitors.

## 1. Introduction

The mesenchymal component of salivary glands plays a pivotal role during development of the salivary gland tissue for induction of epithelial differentiation and branching [1–9]. However, the role of the mesenchymal cells in homeostasis of the adult salivary gland and during repair following injury is understudied. Many salivary gland diseases, if not all, involve and affect the mesenchymal component of the salivary glands, particularly diseases that result in fibrosis of the salivary gland tissue such as radiation-induced damage, Graft-versus-host disease (GVHD), Sjogren's syndrome, and aging [10–14]. In addition, certain salivary gland tumors originate from or involve the mesenchyme [15, 16]. Nonetheless, little attention has been paid in characterizing the salivary gland mesenchymal cells and in developing *in vitro* systems to model the epithelial-mesenchymal interaction during adult homeostasis and disease/injury of the salivary gland tissue. 

We analyzed a transgenic mouse expressing GFP driven by the procollagen promoter (*Col1a1-GFP*) to identify and characterize the mesenchymal cells in the salivary gland tissue. Histological analysis of the salivary glands revealed that only mesenchymal cells of the salivary gland tissue expressed the *GFP* transgene. We tested different culture conditions to *ex vivo* expand mixed cultures of salivary gland mesenchymal and epithelial cells. We determined that medium containing DMEM +10% serum allowed *in vitro* long-term expansion of a mixed culture containing both mesenchymal and epithelial cells. Upon *in vitro* induction on matrigel, this mixed culture differentiated into acini-like structures surrounded by GFP-positive mesenchymal cells. 

TGF-*β*1 signaling is important for development and maintenance of salivary gland tissue but its differential effects on salivary gland epithelium versus mesenchyme have yet not been dissected apart [17–24]. Therefore, we tested the effects of TGF-*β*1 induction and its inhibition in our culture system. TGF-*β*1 induction resulted in elongation of GFP-positive mesenchymal cells, increased collagen production and inhibition of acini-like structure formation. In contrast, inhibition of TGF-*β*R1 resulted in decreased collagen production, increased expression of the mesenchymal fibroblast growth factors, *Fgf-7 *and *Fgf-10*, increased expression of mature acini markers, and formation of larger and more mature acini-like structures. 

This new *in vitro* culture system can be used to expand salivary gland mesenchymal and epithelial cells for tissue regeneration and also to *in vitro* study the role of mesenchymal cells in salivary gland differentiation and alterations of the mesenchymal-epithelial interactions in disease. 

## 2. Materials and Methods

### 2.1. Isolation of Submandibular Salivary Gland Cells

*Col1a1*-*GFP* mice were a kind donation from Dr. Jeremy Duffield [25, 26]. Submandibular salivary gland (SMG) tissues were dissected (one gland per mouse) from 3-month-old *Col1a1*-*GFP* mice (*n* = 3 different preparations) in accordance with approved Institutional Animal Care and Use Committee (IACUC) guidelines, University of Washington. The SMG was separated from the cervical fascia and connective tissue, then gently isolated and kept in phosphate buffer saline (PBS) (Corning Cellgro). The tissues were washed with PBS, mechanically minced with a pair of curved scissors, and enzymatically dissociated with a 1.2 units/mL dispase II, 2 mg/mL collagenase type IV (Worthington) supplemented with 2 mM CaCl_2_ in PBS for 45 min at 37°C. The digested tissues were pipetted up and down several times every 15 min to break up clumps and release mononuclear cells. Subsequently, an equal volume of Dulbecco's modification of eagle's medium (DMEM) with 4.5 g/L glucose, L-glutamine, and sodium pyruvate (Cellgro) was added to the digest prior to filtering through 70 mm nylon cell strainers (BD Falcon) and then centrifuging at 300 g for 10 min at room temperature. The mononuclear cells were then resuspended in two types of growth media described below, and single cell suspensions were initially plated at 50,000 cells/cm^2^ on plastic tissue culture dishes (BD Biosciences).

### 2.2. Culture of Submandibular Salivary Gland Cells

Cells (50,000 cells/cm^2^) were cultured at 37°C under 5% CO_2_ in two kinds of culture media to determine their difference in cell growth, DMEM medium plus 10% heat-inactivated fetal calf serum (HyClone), 100 units/mL penicillin with 100 mg/mL streptomycin (HyClone), and N2 medium containing DMEM, penicillin, streptomycin, 20 ng/mL EGF (Sigma), 20 ng/mL bFGF (Shenandoah biotechnology), 1/100 N2 supplement (Gibco, Invitrogen), 10 *μ*g/mL insulin-transferrin-selenium (ITS) (Cellgro), and 1 *μ*M dexamethasone (Sigma). Fresh medium was added or changed every three days. Once adherent cells were more than 70% confluent, they were detached with 0.25% trypsin-EDTA (Cellgro) and replated at a 1 : 4 dilution under the same culture condition with fresh media. For cells cultured in N2 medium, since they formed spheres, before trypsinization, spheres were collected and then mixed with trypsinized cells before replating in the same condition.

### 2.3. *In Vitro* Differentiation of Submandibular Salivary Gland Cells on Matrigel

Mixed SMG cells (line 1; passage 9; 5 × 10^4^ cells per well) were seeded in either noncoated cells or matrigel-coated plastic surfaces as undifferentiated or differentiated cells, respectively, with 300 *μ*L of additional DMEM media plus serum. Growth factor-reduced matrigel (20 mg/mL; BD Biosciences) was thawed on ice and diluted in PBS at a final concentration of 2 mg/mL. To form three-dimensional matrix in culture dishes, 150 *μ*L of matrigel was added to 48-well tissue culture plate (0.75 cm^2^ per well), incubated at 37°C for 1 hour, and the matrigel excess was removed before cell seeding. Culture media were changed every three days. For differentiation, mixed SMG cells cultured on matrigel were divided into three treatment groups: (1) matrigel alone (control group), (2) transforming growth factor-beta1 (TGF-*β*1; cell signaling; 10 ng/mL), and (3) TGF-*β*1 plus TGF-*β* receptor 1 inhibitor (SB525334; Selleck Chemicals; 1 *μ*M) (TGF-*β*1 + SB525334). At day 3 and 5 of differentiation, both undifferentiated and differentiated cells were fixed with 4% formaldehyde in PBS for 30 min and washed with three times of PBS to preserve GFP expression. The stained cells were determined for acini-like structure formation and further stained for specific salivary gland epithelial and mesenchymal markers. 

### 2.4. RT-PCR and Q-RT-PCR Analyses

Undifferentiated and differentiated mixed SMG cells were extracted for total RNA by using the total RNA kit (Omega Bio-tek) according to the manufacturer's protocol. Quantity and purity of RNA was determined by 260/280 nm absorbance. First-strand cDNA was synthesized from 1 *μ*g of RNA using the high capacity cDNA synthesis kit from Applied Biosystems per manufacturer's protocols using a randomized primer. RT-PCR and Q-RT-PCR mouse-specific primers were included in Table 1. For RT-PCR, cDNA of undifferentiated cells cultured in different passages (20 ng) was diluted in a final volume of 20 *μ*L per reaction using the Immomix PCR Mastermix from Bioline. PCR was performed using the following thermal cycling conditions; 95°C 7 min for initial activation followed by 95°C/30 s; 57°C/30 s; 72°C/45 s, for 35 cycles, with a final 5-min extension at 72°C. Glyceraldehyde-3-phosphate dehydrogenase (*Gapdh*) was utilized as control housekeeping gene. RNA extracted from mouse submandibular salivary gland (SMG) was used as positive controls while negative controls lacked cDNA. For Q-RT-PCR, cDNA of undifferentiated cells cultured in different growth media and differentiated cells (10 ng) was prepared using the Maxima SYBR Green/ROX qPCR master mix (Thermo Scientific). Reactions were processed by the ABI 7900HT PCR system with the following parameters: 50°C/2 min and 95°C/10 min, followed by 40 cycles of 95°C/15 s and 60°C/1 min. Results were analyzed using SDS 2.3 software, and relative expression was calculated using the comparative Ct method. Each sample was run in triplicate reactions for each gene. 

### 2.5. Histology and Staining of Submandibular Salivary Gland Tissues

Submandibular salivary glands (SMGs) were isolated from 3-month-old *Col1a1-GFP* mice (*n* = 3) and removed surrounding connective tissues. To preserve GFP, *Col1a1-GFP* derived SMG was fixed with 4% formaldehyde/PBS for 2 h at RT and washed. The first wash was 30 min followed by 20-min and 10-min washes, respectively. After washing, the fixed SMG was immersed through a gradient of sucrose solutions (10% for 20 min, 20% for 20 min, and 30% for overnight) at 4°C to preserve tissue morphology before embedding in OCT media (VWR) and frozen with liquid nitrogen cooled isobutane. The frozen SMG tissues were cut into 10 *μ*m thickness to get a good morphology of tissue section. Fixed sections were rehydrated and permeabilized with 1% BSA in 0.1% Triton X-100 (Sigma)/PBS for 10 min. Then, sections were blocked with 10% normal goat serum for 1 h at RT and incubated overnight at 4°C with primary antibodies listed in Table 2, following three wash steps. Stained sections were subsequently incubated with goat-derived Alexa 594-conjugated secondary antibody (Invitrogen) at 1 : 1000 dilutions for 1 h at RT, following three times of washing. The tissues were stained with 4′, 6-diamine-2-phenylindol (DAPI) (Life Technologies) at 1 : 1000 to visualize the nuclei. 

### 2.6. Staining of Submandibular Salivary Gland Cells

For immunocytochemistry, undifferentiated and differentiated cells were fixed with 4% formaldehyde in PBS for 30 min and washed three times of PBS. Fixed cells were permeabilized with 1% BSA in 0.1% Triton-X 100/PBS for 10 min and blocked nonspecific binding sites with 10% goat normal serum (Vector Burlingame, CA) for 1 h. All primary antibodies listed in Table 2 were used and incubated overnight at 4°C. Stained cells were incubated with goat-derived Alexa 594-conjugated secondary antibodies (Invitrogen) which were diluted at 1 : 1000 and incubated for 1 h. Cells were stained with DAPI at 1 : 1000 to visualize the nuclei. All antibodies were diluted in 1% BSA in 0.1% Triton-X 100/PBS. IgG isotype from the species made for the primary antibody (0.1 *μ*g/mL) (Vector Burlingame, CA) was used as negative control for all staining. All immunofluorescence images described in this manuscript was detected using either a Zeiss Axiovert 200 fluorescent microscope (Thornwood, NY) or a Nikon A1R Confocal microscope. Photomicrographs were taken with an onboard monochrome AxioCam MRm camera and colored using Adobe Photoshop (San Jose, CA). Background was reduced using brightness and contrast adjustments, and color balance was performed to enhance colors. All the modifications were applied to the whole image using Adobe Photoshop. 

### 2.7. Statistical Analysis

Data of Q-RT-PCR analyses were represented as ± the standard error of the mean (SEM) of results from three separated experiments. The data were analyzed by Student's *t*-test where ***P value ≤ 0.001, **P value ≤ 0.005, or *P value ≤ 0.05 represented significant differences between different culture media or treatments.

## 3. Results

### 3.1. The *Col1a1-GFP* Transgenic Mouse Selectively Identifies Mesenchymal Cells in the Salivary Glands

**Figure 1:**
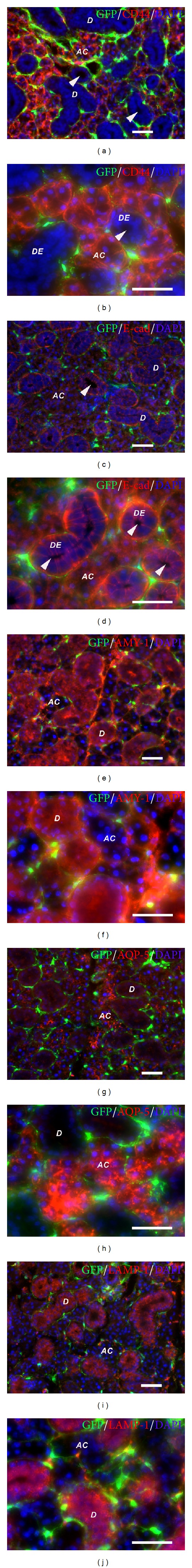
Histology of *Col1a1*-*GFP* derived submandibular salivary gland demonstrates salivary gland epithelial markers. The sections showed that a majority of cells, namely, parenchymal cells in salivary gland tissues were GFP-negative cells, representing salivary gland epithelium (acini and ducts). GFP-positive cells (in green) were anatomically localized in the position of salivary gland mesenchyme and identified as supporting or stromal cells surrounding salivary gland acini or ducts. Salivary gland ducts (D) were anatomically identified as structures with lumens (indicated by arrowheads). (a)–(j), CD44, E-cadherin (E-cad), amylase-1 (AMY-1), aquaporin-5 (AQP-5), and lysosomal-associated membrane protein-1 (LAMP-1) (in red) stained specifically salivary gland epithelium but not mesenchyme. Salivary gland acini (AC) but not salivary gland ductal epithelium (DE) was positive for CD44 (a and b) whereas both salivary gland acini and ductal epithelium were positive for E-cad (c and d). AMY-1 staining was positive in salivary gland acini and particularly strongly positive in ductal epithelium (e and f). Apical and lateral membranes of salivary gland acini were strongly positive for AQP-5 (g and h). LAMP-1 staining was strongly positive in salivary gland ductal epithelium (i and j). Scale bars = 100 *μ*m.

**Figure 2:**
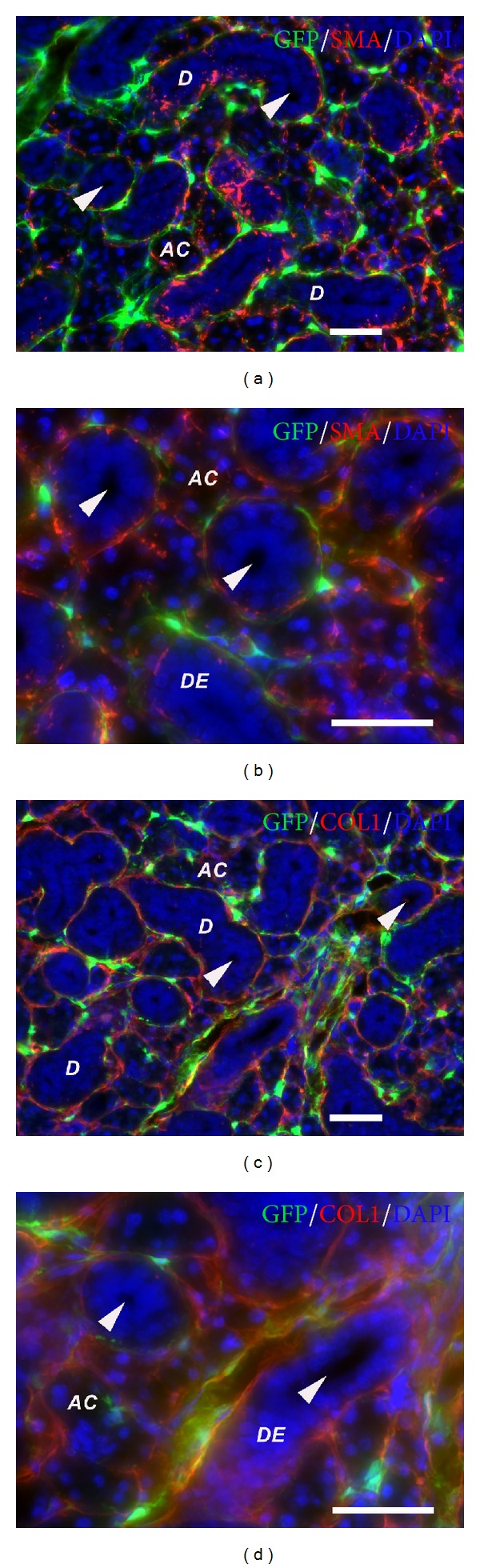
Histology of *Col1a1*-*GFP* derived submandibular salivary gland demonstrates salivary gland mesenchymal markers. (a) and (b), smooth muscle actin (SMA) (in red) staining for myoepithelial cells surrounding acini and ductal structures demonstrated that SMA-positive cells were located closely to, but did not colocalized with, GFP-positive cells. (c) and (d), collagen type I (in red) stained the extracellular matrix in salivary glands, and also colocalized with GFP-positive cells, confirming that GFP expression was driven by the procollagen type I promoter. AC = salivary gland acini, D = salivary gland duct, DE = ductal epithelium. Arrowheads indicate salivary gland ducts (D) with lumens. Scale bars = 100 *μ*m.

 In this study, we analyzed GFP expression in the submandibular salivary glands of *Col1a1*-*GFP* transgenic mice. The *Col1a1*-*GFP* mice express enhanced green fluorescent protein gene under the control of the procollagen, type 1, alpha 1 (*Col1a1*) promoter. We hypothesized that salivary gland mesenchymal cells, but not epithelial cells, express *Collagen type I (Col1a1)* and drive the expression of GFP, resulting in labeled mesenchymal cells by green fluorescence. The histological analysis demonstrated that salivary gland mesenchymal stroma was GFP-positive whereas salivary gland parenchyma or epithelium was GFP-negative (Figure 1). To confirm the specificity of the *Col1a1*-*GFP* mouse model and distinguish differences between salivary gland epithelium and mesenchyme, we stained for markers specific of salivary gland epithelium, CD44, E-cadherin (E-cad), amylase (AMY-1), aquaporin-5, and LAMP-1. CD44 is a cell surface glycoprotein found on basal epithelial cells including salivary gland epithelium [27, 28]. CD44 staining was positive in basal and lateral membranes of salivary gland acini (AC) but not in ductal epithelium (DE) and mesenchyme (Figures 1(a) and 1(b)). E-cadherin (epithelial-calcium-dependent adhesion or E-cad) is a transmembrane protein which is crucial for cell-cell interaction in organ development including salivary gland formation, and expressed by salivary gland epithelium [29]. E-cadherin staining was positive for both salivary gland epithelial cells of acini and ducts (D) in particular at acinar cell-ductal cell contacts but not mesenchyme (Figures 1(c) and 1(d)). Salivary amylase catalyses the breakdown of starch into sugars and is found in the granular convoluted tubular cells and to a lesser extent in the acinar cells of submandibular gland (Figures 1(e) and 1(f)) [30]. Aquaporin 5 is important for fluid transport and saliva secretion and is found in luminal, lateral, and basal membrane of acinar cells (Figures 1(g) and 1(h)) [31]. LAMP-1 is a lysosome-associated protein found in the ductal and acinar cells in salivary gland (Figures 1(i) and 1(j)) [32]. Smooth muscle actin (SMA) staining for myoepithelial cells [29] exhibited no colocalization between SMA and GFP found in mesenchymal cells, illustrating a differential staining pattern between mesenchymal and myoepithelial cells in normal salivary glands (Figures 2(a) and 2(b)). Additionally, staining for collagen type I mostly colocalized with GFP-positive mesenchymal cells, confirming the specificity of the *Col1a1-GFP* mouse model. As expected, we also observed positive collagen type I staining in salivary gland extracellular matrices labeling the basement membrane which was not positive for GFP as it was not cytoplasmic but extracellular collagen (Figures 2(c) and 2(d)). 

### 3.2. Mixed Salivary Gland Cells Cultured in Different Media Exhibited Differential Growth of Salivary Gland Epithelial and Mesenchymal Cells

**Figure 3:**
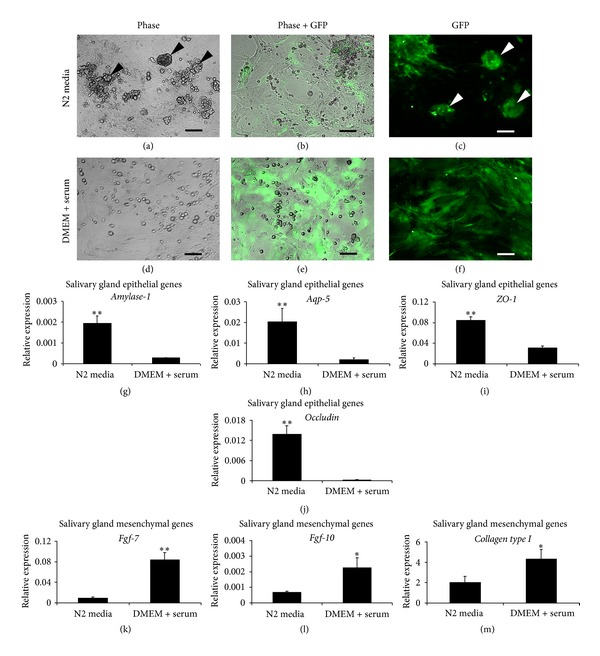
*In vitro* culture of *Col1a1-GFP* derived submandibular salivary gland cells. Mixed salivary gland epithelial and mesenchymal cells (passage 1, for 2 weeks) exhibited different growth pattern and morphology when cultured in N2 media versus DMEM plus serum medium. (a)–(c) Mixed salivary gland cells were cultured in N2 media. A majority of cells grown in N2 media were polyhedral-shaped and GFP-negative, representing salivary gland epithelial cells (a and b). Some GFP+ mesenchymal cells were also found in this culture condition (b and c). N2 media enhanced sphere formation containing both salivary gland epithelial and mesenchymal cells (indicated by arrowheads) (a and c). (d)–(f) DMEM plus 10% serum promoted the growth of salivary gland mesenchymal cells which were shown as spindle-shaped and GFP-positive cells. Small round and GFP-negative cells were also observed on top of the mesenchymal or stromal monolayer, indicating the existence of salivary gland epithelial cells (d and e). (g)–(m) Quantitative specific gene expression was analyzed to confirm the presence of salivary gland epithelium and mesenchyme in both N2 and DMEM media plus serum. The expression of salivary gland epithelial genes, *Amylase-1* (g), *Aquaporin-5* (*Aqp-5*) (h), *Zonula occludens-1* (*ZO-1*) (i), and *Occludin* (j), were significantly upregulated in N2 media-cultured cells. The expression of salivary gland mesenchymal genes, *Fgf-7* (k), *Fgf-10* (l), and *Collagen type I* (m), significantly increased in cells cultured in DMEM plus serum medium. Relative expression was normalized to the expression of *Gapdh* which was used as the reference gene. Values were represented as mean ± SEM from three independent experiments (*n* = 3). Student's *t*-test was analyzed to compare between cells cultured in N2 and DMEM media plus serum, **P ≤ 0.005 and *P ≤ 0.05. Scale bars = 100 *μ*m.

 To select a culture condition system capable of promoting proliferation of mixed cell populations containing salivary gland epithelium and mesenchyme, we used two different kinds of media to culture mixed salivary gland cells, N2 medium and DMEM plus 10% serum. N2 medium contains DMEM supplemented with EGF, bFGF, N2, and ITS and has been used as growth and differentiation media for salivary gland stem cells [33]. DMEM plus 10% serum has previously been used as medium to promote salivary gland epithelial and mesenchymal cell growth [34, 35]. After 2 weeks in culture (cell passage 1), in N2 medium we observed sphere formation combined with monolayer of polyhedral- and spindle-shaped cells (Figure 3(a), arrowheads). The monolayer cells were mainly negative for GFP (Figure 3(b)). Most of the spheres formed were negative for GFP but some of them were also GFP-positive (Figure 3(c), arrowheads). This indicates that N2 medium induces both salivary gland epithelium and mesenchyme to form spheres, and a majority of cells proliferating in N2 medium were epithelial cells. However, mixed salivary gland cells in this N2 medium failed to proliferate beyond 2 weeks of culture and were not able to survive. In contrast, mixed cells cultured in DMEM plus serum did not form spheres but grew as polyhedral-shaped or round cells on top of spindle-shaped monolayer cells (Figure 3(d)). The major cell population was spindle-shaped cells expressing GFP (Figures 3(e) and 3(f)). This cell mixture was able to proliferate and graw beyond passage 10 (2 month in culture). Interestingly, after several cell passages, both spindle-shaped and polyhedral-shaped cells formed clusters of monolayer cells. The former showed cells that were GFP-positive while the latter were GFP-negative. Thus, the DMEM plus serum medium effectively enhanced the proliferation of both salivary gland epithelial and mesenchymal cells even in late passages. To confirm the presence of epithelial and mesenchymal cells in our cultures, Q-RT-PCR was performed to compare the level of salivary gland epithelial and mesenchymal gene expression between cells cultured in N2 versus DMEM media (Figures 3(g)–3(m)). As expected, mixed cells cultured in N2 medium significantly upregulated all salivary gland epithelial genes, *Amylase-1*, *Aqp-5*, *ZO-1*, *Occludin *(*P* ≤ 0.005), and downregulated all salivary gland mesenchymal genes, *Fgf-7 *(*P* ≤ 0.005), *Fgf-10 *(*P* ≤ 0.05), and *Collagen type I *(*P* ≤ 0.05). In contrast, DMEM plus serum seems to support salivary gland mesenchymal cell growth as evidence by increased gene expression levels of mesenchymal genes. Based on the culture morphological analysis and gene expression analysis the N2 medium enhanced more growth of salivary gland epithelium than mesenchyme but was only able to sustain these cells for a short period whereas the DMEM plus serum effectively promoted the proliferation of both salivary gland epithelium and mesenchyme in long-term culture. Therefore, we selected to use DMEM plus serum as the expanding culture medium for all the further experiments described herein. 

### 3.3. DMEM Plus Serum Medium Enhanced *In Vitro* Long Term Proliferation and Maintenance of Salivary Gland Mesenchymal and Epithelial Cells

**Figure 4:**
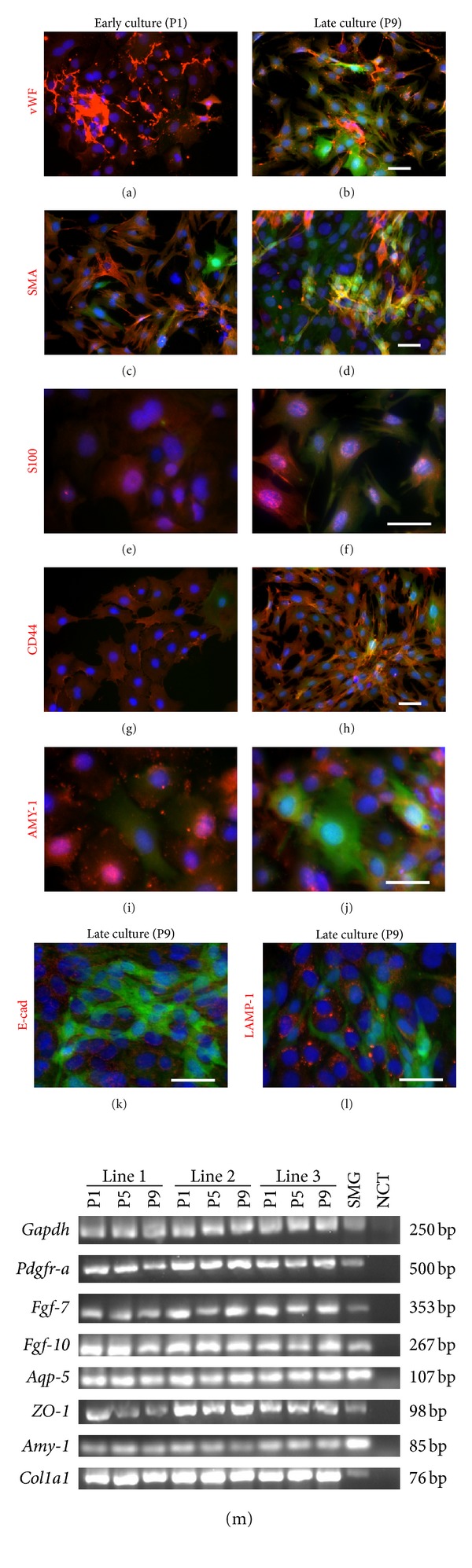
Characterization of *Col1a1-GFP* derived submandibular salivary gland cells cultured in DMEM plus serum media. (a)–(l) Specific staining showed the presence of salivary gland epithelial and mesenchymal cells in DMEM media plus serum in early (passage 1; 1 week in culture) and late cultures (passage 9; 8 weeks in culture). GFP-positive spindle-shaped cells represented mesenchymal cell population. Cells in early culture were stained positively for von Willebrand Factor (vWF) (a), smooth muscle actin (SMA) (c) and S100 (e), CD44 (g), and amylase-1 (AMY-1) (i) (in red), which are markers for endothelial cells, myoepithelial cells, and salivary gland epithelial cells, respectively. In the late culture, an increased number of GFP-positive cells was observed. vWF (b), SMA (d), S100 (e), and CD44 (h) staining were seen. SMA, S100, and CD44 expression seem to be increased in the late passaged mesenchymal cells, which was illustrated by the costaining of SMA, S100, and CD44 with GFP. SMA staining demonstrated four cell populations in mixed salivary gland cultured cells, GFP+/SMA+, GFP+/SMA−, GFP−/SMA+, and GFP−/SMA− cells but a majority of cells were GFP+/SMA− cells, indicating some mesenchymal cells upregulated SMA expression in the late culture (d), compared to that in the early culture (c). AMY-1 (j), E-cadherin (E-cad) (k), and LAMP-1 (l) were specifically positive for salivary gland epithelium in red, but not mesenchymal cells. (f) RT-PCR analysis displayed a gene profile corresponding of a mixed salivary gland cell culture throughout long-term culture in DMEM plus serum medium from early through late passages. The gene expression of salivary gland epithelium, *Aqp-5 (Aquaporin-5)*, *ZO-1 (Zona occludens-1)*, and *Amy-1 (Amylase-1)*, as well as salivary gland mesenchyme, *Pdgfr-a*, *Fgf-7*, *Fgf-10*, *Col1a1 (Collagen type I)*, were detected in both early and late passages, indicating the existence of salivary gland epithelial and mesenchymal in these cultures. This gene expression profile was detected in three different lines of salivary gland cells which were derived from three different *Col1a1*-*GFP* mice. Early culture = passage 1 (P1), and late culture = passage 9 (P9). P1, 5, and 9 = passages 1, 5, and 9, respectively. Submandibular salivary gland (SMG) was used as positive control whereas no template was used as negative control (NCT). Scale bars = 100 *μ*m.

 Mixed salivary gland cells cultured in DMEM plus serum medium proliferated for at least 10 passages (approximately 2 months in culture) without alterations in morphology and proliferation rate. In each passage, the presence of GFP-positive cells and GFP-negative cells was monitored. Two cell types were consistently observed in each passage: a majority of spindle-shaped cells expressing GFP and GFP-negative polyhedral-shaped cells, representing salivary gland mesenchymal and epithelial cells, respectively (Figures 4(a)–4(e)). In early passage we detected several cell types in addition to GFP+ mesenchymal cells and epithelial cells. von Willebrand Factor (vWF) is expressed by microvascular endothelial cells of multiple tissues, including the salivary gland [36]. We detected vWF+ endothelial cells in early and late cultures (Figures 4(a) and 4(b)). As shown in Figures 2(a) and 2(b), SMA is expressed by myoepithelial cells in the salivary gland tissue. We detected SMA+ cells in early and late cultures that were GFP negative (Figures 4(c) and 4(d)). Interestingly, we observed that some GFP+ cells are also SMA+ in late culture suggesting that some GFP+ mesenchymal cells can upregulate SMA expression (Figure 4(d)). S100 is another marker expressed by myoepithelial cells [37]. We detected S100+ cells in early and late cultures (Figures 4(e) and 4(f)). Interestingly, some GFP+ cells costained positive for S100 (Figure 4(f)). Epithelial cells expressed CD44 in early and late cultures (Figures 4(g) and 4(h)). However, we also observed that some GFP+ cells coexpressed CD44 in late cultures (Figure 4(h)). Epithelial cells expressed amylase (AMY-1) in early and late cultures (Figures 4(i) and 4(j)). Epithelial cells in late cultures also expressed E-cad, and lysosomal associated membrane protein (LAMP-1) [38] (Figures 4(k) and 4(l)). In addition, we examined the gene expression of salivary gland epithelium, *Aqp-5*, *ZO-1*, *Amy-1*, and mesenchyme, *Pdgfr-a*, *Fgf-7, Fgf-10, Col1a1,* in cells from different passages (passages 1, 5, and 9) to confirm whether the cultured cells in the late passage still contained two cell populations. The gene profile by RT-PCR demonstrated the existence of both salivary gland epithelial and mesenchymal cells in the late passage of our culture (Figure 4(m)). 

### 3.4. Expression of TGF-*β*1 Ligand and Receptor in Salivary Gland Tissues and Cultured Cells

**Figure 5:**
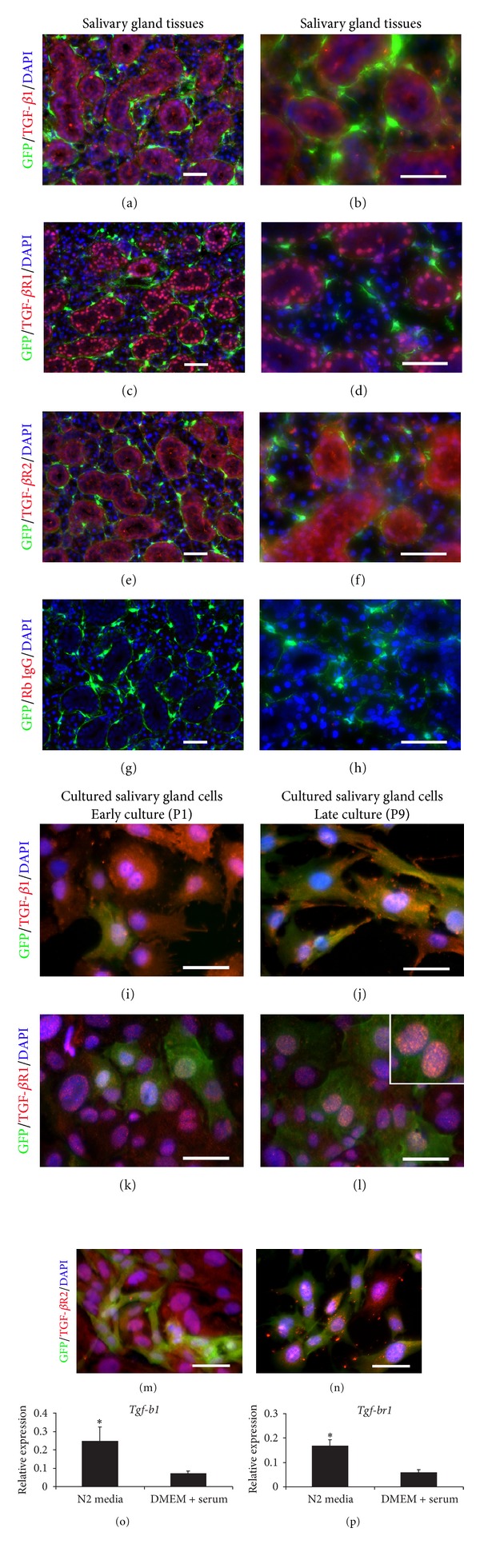
The expression of TGF-*β*1 ligand and receptors in *Col1a1-GFP* derived submandibular salivary gland tissues and cultured cells. (a)–(h) Immunofluorescence of *Col1a1*-*GFP* derived submandibular salivary gland tissues taken by Zeiss fluorescence microscope. Salivary gland epithelium, in particular ductal epithelium, strongly expressed TGF-*β*1 (in cytoplasm) (a and b), TGF-*β* receptor 1 (TGF-*β*R1) (in nuclei and cytoplasm) (c and d), and TGF-*β* receptor 2 (TGF-*β*R2) (in nuclei and cytoplasm) (in red) (e and f). GFP-positive salivary gland mesenchyme (in green) did not seem to highly express either TGF-*β*1 or TGF-*β*R1, in normal salivary gland tissues. (e) and (f) Rabbit IgG control was used as negative control to confirm the specificity of TGF-*β*1, TGF-*β*R1, and TGF-*β*R2 antibodies. (i)–(n) Cultured salivary gland cells in DMEM plus serum medium from early (passage 1) and late (passage 9) cultures showed the different expression of TGF-*β*1, TGF-*β*R1, and TGF-*β*R2. In the early culture, TGF-*β*1 and TGF-*β*R2 were strongly expressed in the cytoplasm of epithelial cells (i and m, resp.) whereas the strong TGF-*β*R1 expression was found in cultured salivary gland epithelial cells in cytoplasmic and nuclear areas (k), but not in mesenchymal cells. In the late culture, the expression of TGF-*β*1, TGF-*β*R1, and TGF-*β*R2 was seen in both salivary gland epithelial and mesenchymal cells (j, l, and n). The salivary gland mesenchymal cells increased the expression of TGF-*β*1 (j), TGF-*β*R1 (l, inset), and TGF-*β*R2 (n) after late culture. The staining pattern of TGF-*β*1 and TGF-*β*R1 in late cell passage was similar to early cell passage (j and l) whereas that of TGF-*β*R2 was shown in both membrane and nuclei (n). (o) and (p) Mixed salivary gland cells cultured in N2 media which contained a majority of salivary gland epithelium showed higher levels of both *Tgf-b1 *and *Tgf-br1* expression, compared to that in DMEM media plus serum. Relative expression was normalized to the expression of *Gapdh *which was used as the reference gene. Values were represented as mean ± SEM from three independent experiments (*n* = 3). Student's *t*-test was analyzed to compare between cells cultured in N2 and DMEM media plus serum, *P ≤ 0.05. Scale bars = 100 *μ*m.

We then examined the expression of TGF-*β*1 ligand (TGF-*β*1) and its receptors, TGF-*β*1 receptor 1 (TGF-*β*R1) and receptor 2 (TGF-*β*R2), in both salivary gland tissues and cultured cells. Murine submandibular salivary gland tissues showed the expression of TGF-*β*1, TGF-*β*R1, and TGF-*β*R2 in salivary gland epithelium, particularly in ductal epithelium (Figures 5(a)–5(f)). However, we observed very low expression of TGF-*β*1 and TGF-*β*R1 in the salivary gland mesenchyme. In contrast, some mesenchymal cells (GFP)+ in the salivary gland stained positive for TGF-*β*R2 (Figures 5(e) and 5(f)). As negative control, staining of salivary gland tissues with rabbit IgG control exhibited completely negative staining result used to confirm the specificity of TGF-*β*1, TGF-*β*R1, and TGF-*β*R2 antibodies (Figures 5(g) and 5(h)). We then studied the protein expression of TGF-*β*1 and its receptors in early and late cultures. Salivary gland epithelial cells stained strongly positive for TGF-*β*1 and TGF-*β*R1 in early cultures. Interestingly, in early cultures mesenchymal cells, which were GFP-positive, showed weak positive staining for TGF-*β*1 and its receptors (Figures 5(i), 5(k), and 5(m)). In contrast, in late cultures GFP+ mesenchymal cells stained strongly positive for TGF*β*1 and its receptors (Figures 5(j), 5(l), and 5(n)). In particular, cultured salivary gland mesenchymal cells showed stronger TGF-*β*R1 expression than that in epithelial cells (Figure 5(l)). The TGF-*β*R1 antibody we used recognizes the cytoplasmic domain which can be cleaved and can translocate to the nucleus [39]. This explains why we observed mainly nuclear staining. Q-RT-PCR analysis of mixed salivary gland cells from passage 2 demonstrated that the N2 medium which contained a majority of epithelial cells showed increased levels of *Tgf-b1* and *Tgf-br1*, compared to DMEM plus serum (*P* ≤ 0.05) (Figures 5(o) and 5(p)). 

**Figure 6:**
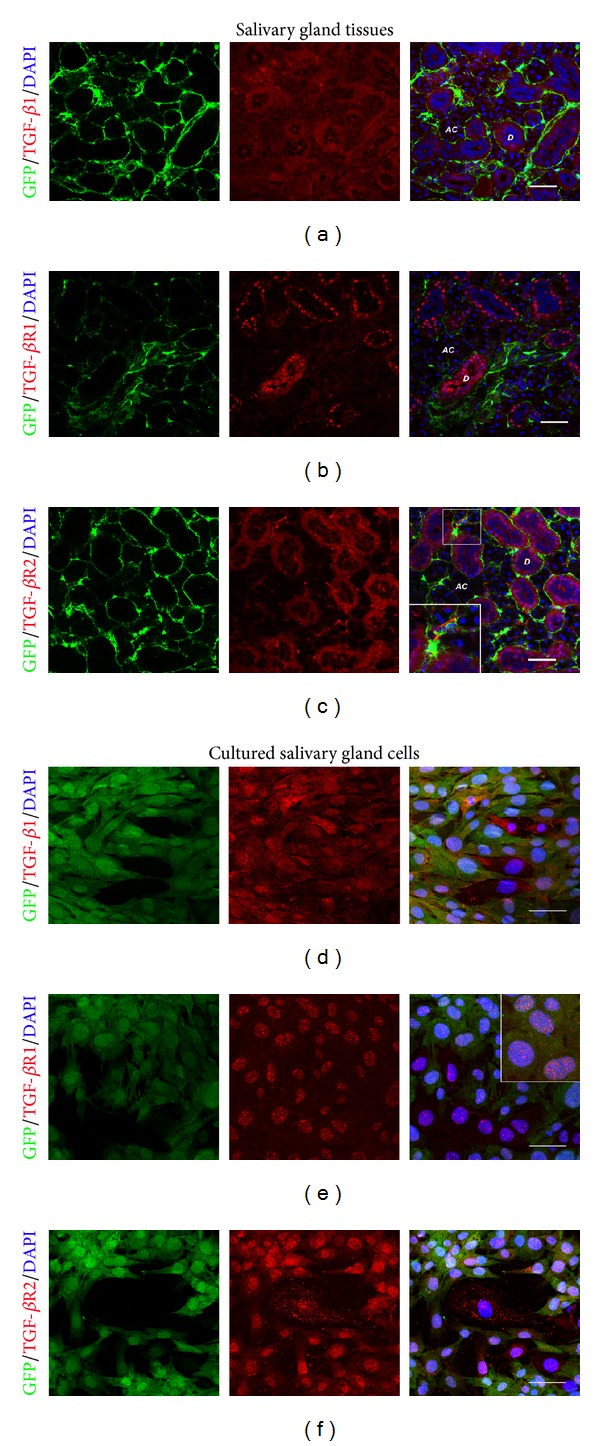
Confocal microscopy allows clear visualization of the differential protein expression of TGF-*β*1 ligand and receptors in *Col1a1-GFP* derived submandibular salivary gland tissues and cultured cells. (a)–(c) Salivary gland epithelium, in particular ductal epithelium, strongly expressed TGF-*β*1 (in cytoplasm) (a), TGF-*β* receptor 1 (TGF-*β*R1) (in nuclei and membrane) (b), and TGF-*β* receptor 2 (TGF-*β*R2) (in nuclei and membrane) (in red) (c). GFP-positive salivary gland mesenchyme (in green) did not seem to highly express either TGF-*β*1 or TGF-*β*R1  in normal salivary gland tissues. However, some GFP+ mesenchymal cells were strongly positive for membranous TGF-*β*R2 staining in normal salivary gland tissues (c and inset). (d)–(f) Both cultured salivary gland epithelial and mesenchymal cells in late cultures (passage 9) showed the expression of TGF-*β*1, TGF-*β*R1, TGF-*β*R2. The salivary gland mesenchymal cells increased the expression of TGF-*β*1 (d), TGF-*β*R1 (e, inset), and TGF-*β*R2 (f) after late culture. The staining pattern of TGF-*β*1 was seen in cytoplasm (d) whereas those of TGF-*β*R1 and TGF-*β*R2 were shown in both membrane and nuclei (e, inset, and f). AC = salivary gland acini, D = salivary gland duct. Scale bars = 50 *μ*m.

 The regulation TGF-*β* signaling pathway at the receptor level is well understood. Briefly, TGF-*β* ligands (TGF-*β*1, 2, 3) bind TGF-*β*R2 which recruits TGF-*β*R1 to form a heterotetramer (two type I and two type II receptors) [40]. The formation of this heterotetramer is needed for TGF-*β* signaling. TGF-*β*R2 phosphorylates TGF-*β*R1, activating TGF-*β*R1 kinase which mediates the Smad pathway. We observed membranous and nuclear staining of TGF-*β*R1 and TGF-*β*R2 on GFP+ mesenchymal cells, indicating that the TGF-*β* signaling is active in cultures, especially in late cultures. To confirm the staining pattern for TGF-*β* and its receptors, we performed fluorescence image analysis by confocal microscopy. Similar to the results presented in Figure 5, in the salivary gland tissue, TGF-*β*1 and TGF-*β*R1 is expressed mainly by epithelial cells (Figures 6(a) and 6(b)), whereas TGF-*β*R2 is expressed by epithelial cells but also strongly expressed by GFP+ mesenchymal cells (Figure 6(c), inset). In contrast, in late cultures TGF-*β*1 and its receptors are expressed by both epithelial cells and GFP+ mesenchymal cells (Figures 6(d)–6(f)), indicating an up-regulation of TGF-*β*1 and its receptors in cultured GFP+ cells, perhaps mediated by a positive feedback driven by the TGF-*β*1 produced by the epithelial cells early in culture (Figure 5(i)). 

### 3.5. TGF-*β*R1 Inhibitor Promoted the *In Vitro* Formation of Acinar-Like Structures

**Figure 7:**
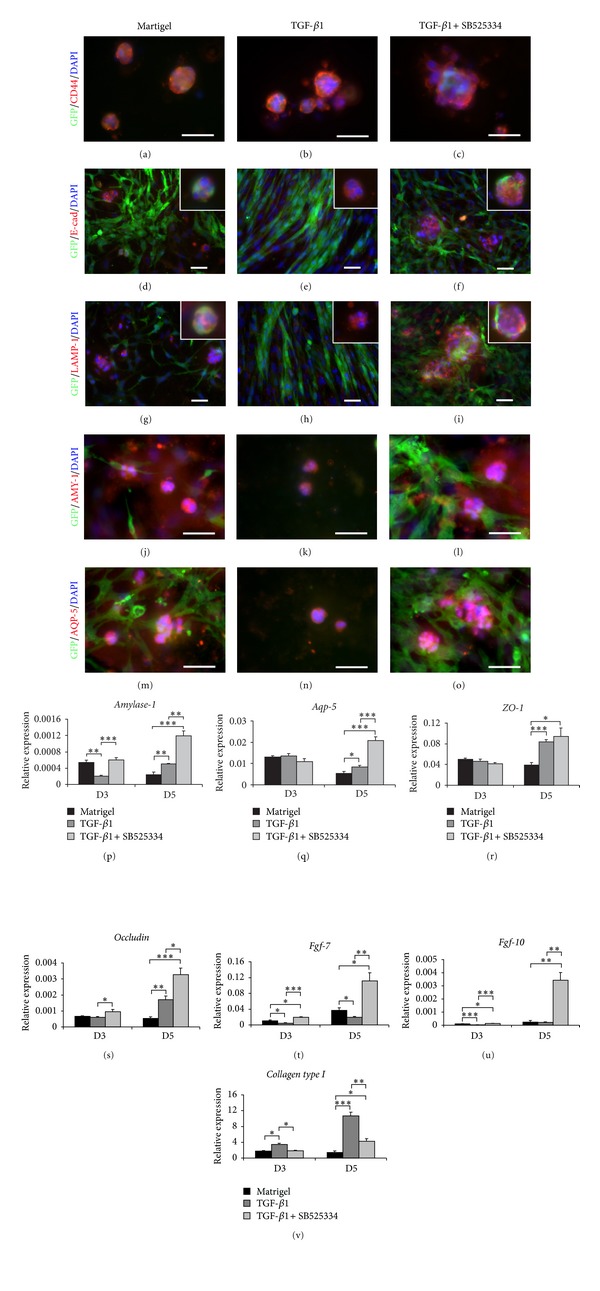
*In vitro* differentiation of *Col1a1-GFP* derived submandibular salivary gland cells on matrigel treated with TGF-*β*1 or TGF-*β*1 plus TGF-*β*R1 inhibitor, SB525334 (TGF-*β*1 + SB525334). Mixed cells were plated on matrigel with DMEM plus serum medium alone (left column) or supplemented with TGF-*β*1 (middle column) or TGF-*β*1 with SB525334 inhibitor (right column). Specific staining for salivary gland epithelial cells demonstrated the presence of differentiated salivary gland cells at day 5. (a)–(c) CD44 staining (in red) showed the formation of acinar-like structures (round structures with polarized nuclei) in all three groups. Large acini-like structures with branching were found in the TGF-*β*1 + SB525334 (c). (d)–(o) Salivary gland epithelium and acinar formation were identified by E-cadherin (E-cad), LAMP-1, AMY-1, and AQP-5 staining (in red). Salivary gland acini-like structures (in red) closely located to mesenchymal cells (in green) were found in the matrigel (d, g, j, and m) and TGF-*β*1 + SB525334 (f, i, l, and o) groups whereas no acinar formation was observed in close proximity of mesenchymal cells in the TGF-*β*1 group (e and h). Acinar-like structures were also found in areas free of mesenchymal cells in all three groups. Cells in the TGF-*β*1 + SB525334 (c, f, i insets, l, and o) formed larger acini-like structures than those in the matrigel alone (a, d, g insets, j, and m) and TGF-*β*1 (b, e, h insets, k, and n). Salivary gland mesenchymal cells were polarized and elongated in the TGF-*β*1 (e and h) whereas mesenchymal cells in the matrigel (d, g, and m) and TGF-*β*1 + SB525334 (f, i, l, and o) demonstrated cobblestone appearance. Some GFP-positive cells were found to integrate or locate peripherally to acini-like structures in the matrigel (a, d, and g insets) and TGF-*β*1 + SB525334 (c, f, and i insets), but not in the TGF-*β*1 (b, e, and h insets). (j)–(p) The expression of salivary gland epithelial genes, *Amylase-1*, *Aquaporin-5 *(*Aqp-5*), *Zonula occludens (ZO-1)*, *Occludin*, and salivary gland mesenchymal genes, *Fgf-7*, *Fgf-10*, and *Collagen type I*, was determined after 3- and 5-day treatments. At day 3 (D3), cells treated with TGF-*β*1 + SB525334 significantly upregulated some of salivary gland epithelial genes, *Amylase-1* and *Occludin*, and all salivary gland mesenchymal genes except *Collagen type I*, compared to cells on matrigel alone and/or cells treated with TGF-*β*1. The TGF-*β*1-treated cells significantly expressed lower level of *Amylase-1 *expression (p), but higher level of *Collagen type I *(v), compared to untreated cells, and TGF-*β*1 + SB525334-treated cells. At day 5 (D5), cells treated with TGF-*β*1 + SB525334 expressed the highest levels of both salivary gland epithelial and mesenchymal genes and were significantly different compared to untreated and TGF-*β*1 treated cells. The *ZO-1* expression was comparable but insignificantly different between TGF-*β*1 and TGF-*β*1 + SB525334 treated cells (r). The expression of *Fgf-7* and *Fgf-10* was remarkably increased in the TGF-*β*1 + SB525334 group (t and u). The highest level of *Collagen type I* was observed and shown a significantly statistical difference in TGF-*β*1 treated cells at both day 3 and 5 compared to other groups (v). Relative expression was normalized to the expression of *Gapdh *which was used as the reference gene. Values were represented as mean ± SEM from three independent experiments (*n* = 3). Student's *t*-test was analyzed to compare between Matrigel (untreated; black bar), TGF-*β*1 treated (dark gray bar), and TGF-*β*1 + SB525334 treated cells (light gray bar), ***P ≤ 0.001, **P ≤ 0.005, or *P ≤ 0.05. Scale bars = 100 *μ*m.

 Mixed salivary gland cells (from passage 9) induced on matrigel differentiated into acini-like structures demonstrating that cells maintained in long-term cultures still have differentiation capacity [6, 7]. TGF-*β*1 signaling is important for salivary gland formation during development [18, 21, 23]. To determine the effect of TGF-*β*1 signaling on *in vitro* differentiation of salivary gland cells, we compared the differentiation capacity of matrigel-induced cells exposed to TGF-*β*1 (TGF-*β*1) or TGF-*β*1 plus TGF-*β*R1 inhibitor (TGF-*β*1 + SB525334). SB5255334 is a potent inhibitor of TGF-*β*R1 kinase activity [41, 42]. The *in vitro* differentiation was conducted at two time points between day 3 and 5 and analyzed by specific antibody staining to salivary gland epithelium (at day 5) and the gene expression of salivary gland epithelial and mesenchymal genes (at day 3 and 5). The specific staining of epithelium, CD44 (Figures 7(a)–7(c)), E-cad (Figures 7(d), 7(e) inset, 7(f)), LAMP-1 (Figures 7(g), 7(h) inset, 7(i)), amylase (Figures 7(j)–7(l)), and aquaporin 5 (Figures 7(m)–7(o)) showed that salivary gland epithelial cells plated on matrigel in all three groups were able to differentiate based on the formation of acinar-like structures. As expected, all acinar-like structures found were GFP-negative, suggesting that only salivary gland epithelial cells differentiated into acini-like structures on matrigel (Figures 7(a)–7(c)). In addition, in all the differentiation cultures on matrigel we observed areas of mesenchymal clusters and areas free of mesenchymal cells (shown as figures with empty black backgrounds in Figures 7(a)–7(c), 7(k), and 7(n) and all insets). In control (matrigel only) and TGF-*β*1 + SB525334 cultures, abundant acinar-like structures were found in both mesenchymal rich and mesenchymal free areas whereas in TGF-*β*1 exposed cultures acinar-like structures were observed only in the mesenchymal free areas. Most of the acinar-like structures in control (matrigel only) and TGF-*β*1 + SB525334 cultures were in close proximity to mesechymal cells (Figures 7(d), 7(g), 7(j), and 7(m) and Figures 7(f), 7(i), 7(l), and 7(o)). Moreover, the size of acinar-like structures found in the TGF-*β*1 + SB525334 group (Figures 7(c), 7(f), 7(i), 7(l), and 7(o) and insets) was remarkably larger than that in TGF-*β*1 group (Figures 7(b), 7(e), 7(k), and 7(n), and insets). Interestingly, in the control (matrigel only) (Figures 7(d), 7(j), 7(m), and 7(g), and insets) and TGF-*β*1 + SB525334 groups (Figures 7(f), 7(i), 7(l), and 7(o) and insets), we found GFP-positive cells located peripherally and closely associated with most acinar-like structures, mimicking salivary gland acini *in vivo*, but this association was not found in the TGF-*β*1 group (Figures 7(e) and 7(h) insets). The mesenchymal cells in control (matrigel only) and TGF-*β*1 + SB525334 groups showed a cobblestone-appearance (Figures 7(d), 7(g), 7(m) and 7(f), 7(i), 7(l), 7(o)) whereas that in TGF-*β*1 showed elongation and polarization (Figures 7(e) and 7(h)). However, no difference in number of acini-like structures among three groups was found.

 Q-RT-PCR analysis showed that at day 3 (Figures 7(p)–7(v)), cells in both the control (matrigel only) and TGF-*β*1 + SB525334 groups showed higher levels of salivary gland epithelial and mesenchymal genes, but lower levels of *Collagen type I*, compared to cells in TGF-*β*1. Cells in control group (matrigel only) showed significantly higher levels of *Amylase-1 *(*P* ≤ 0.05), *Fgf-7 *(*P* ≤ 0.005), and *Fgf-10 *(*P* ≤ 0.001) but lower levels of *Collagen type I *(*P* ≤ 0.05), compared to cells in the TGF-*β*1 group. Likewise, cells in the TGF-*β*1 + SB525334 group showed significantly higher levels of *Amylase-1 *(*P* ≤ 0.001), *Occludin *(*P* ≤ 0.05)*, Fgf-7*, and *Fgf-10 *(*P* ≤ 0.001) but lower *Collagen type I *(*P* ≤ 0.05), compared to cells in the TGF-*β*1 group. At day 3, no difference was found in salivary gland epithelial genes expressed by cells in the control (matrigel only) compared to that in the TGF-*β*1 + SB525334 group. In contrast, significantly higher levels of *Fgf-7 *and *Fgf-10 *(*P* ≤ 0.005) were expressed by cells in the TGF-*β*1 + SB525334 group compared to the control group (matrigel only). 

 The comparison of the acinar genes from day 3 to day 5 in the control group (matrigel only) indicates a decreasing trend which suggests that additional supplementation of differentiation factors may be needed to induce progression of differentiation on matrigel. Interestingly, at day 5, cell induced with TGF-*β*1 showed significantly higher levels of all of salivary gland epithelial genes, *Amylase-1* (5 folds, *P* ≤ 0.005), *Aqp-5 *(*P* ≤ 0.05), *ZO-1 *and *Occludin *(4 folds, *P* ≤ 0.005), and *Collagen type I* (10 folds, *P* ≤ 0.001), compared to the matrigel group, whereas *Fgf-7* levels were decreased (*P* ≤ 0.05), compared to the matrigel group. This suggested that TGF-*β*1 may be beneficial for epithelial cell differentiation but inhibited expression of mesenchymal FGFs. Conversely, cells in the TGF-*β*1 + SB525334 group significantly upregulated most of the salivary gland epithelial genes, *Amylase-1 *(3 folds) and *Aqp-5 *(*P* ≤ 0.005), *Occludin *(*P* ≤ 0.05), and mesenchymal genes, *Fgf-7* (10 folds) and *Fgf-10* (15 folds) (*P* ≤ 0.05), but downregulated *Collagen type I *(3 folds, *P* ≤ 0.05), when compared with cells in the TGF-*β*1 group. Interestingly, at day 5 cells treated with TGF-*β*1 + SB525334 upregulated all epithelial and mesenchymal genes including *Collagen type I* when compared with control group (matrigel only), indicating TGF-*β*1 signaling inhibition acts differentially on epithelial and mesenchymal cells. 

### 3.6. TGF-*β*1 Induced Expression TGF-*β*1 Ligand and Its Receptor

**Figure 8:**
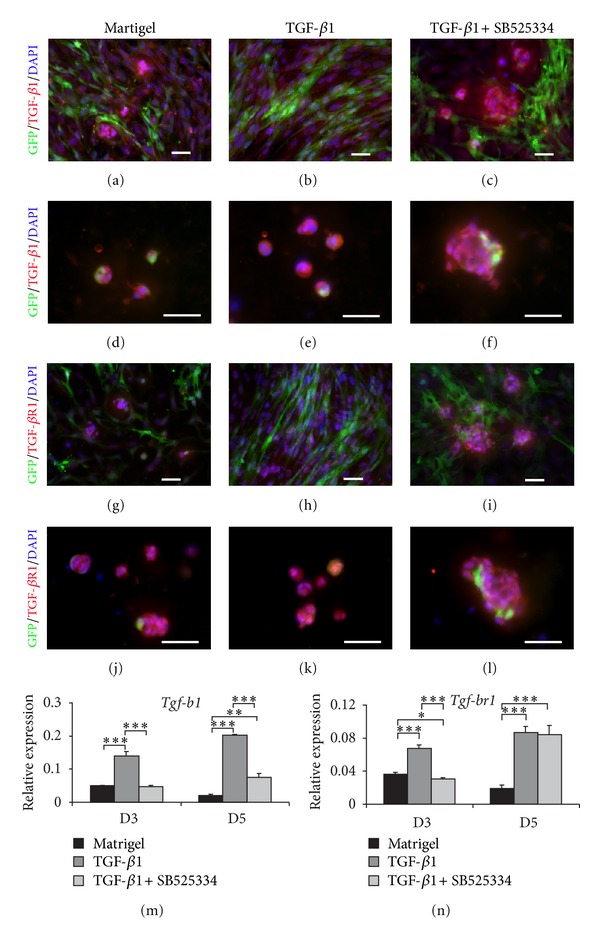
The expression of TGF-*β*1 ligand and receptor in *Col1a1*-GFP derived submandibular salivary gland cells after *in vitro* differentiation on matrigel treated with TGF-*β*1 or TGF-*β*1 and plus TGF-*β*R1 inhibitor, SB525334 (TGF-*β*1 + SB525334). (a)–(f) and (g)–(l) TGF-*β*1 and TGF-*β*1R staining (in red) showed the formation of acinar-like structures, respectively. Acinar formation was observed adjacent to mesenchymal cells in the matrigel only (a and g) and TGF-*β*1 + SB525334 (c and i) groups, but not in the TGF-*β*1 (b and h) group. In the areas free of mesenchymal cells, acinar-like structures were also found in all three groups. TGF-*β*1 + SB525334 treated cells formed larger acinar-like structures (c, f, i, and l) compared to untreated (a, d, g, and j) and TGF-*β*1 treated cells (e and k). Acini-like structures in the matrigel (d and j) and TGF-*β*1 + SB525334 (f and l), but not in the TGF-*β*1 (e and k) showed GFP-positive mesenchymal cells located peripherally or adjacent to acini-like structures. Elongated and polarized salivary gland mesenchymal cells were found in the TGF-*β*1 treated group (b and h), but not in the untreated (a and g) and TGF-*β*1 + SB525334 groups (c and i). (m) and (n) The expression of *TGF- β1 *ligand *(Tgf-b1)* and *receptor (Tgf-br1) *were also examined, respectively. The cells treated with TGF-*β*1 significantly increased the level of *Tgf-b1 *and *Tgf-br1* expression compared to untreated and TGF-*β*1 + SB525334 treated cells both day 3 (D3) and similar pattern was observed at day 5 (D5). In the control, untreated cells on matrigel, the expression levels of both *Tgf-b1* and receptor *Tgf-br1* decreased at D5 compared to D3 but this pattern is not seen in the TGF-*β*1 and TGF-*β*1 + SB525344 groups. Relative expression was normalized to the expression of *Gapdh *which was used as the reference gene. Values were represented as mean ± SEM from three independent experiments (*n* = 3). Student's *t*-test was analyzed to compare between matrigel (untreated; black bar), TGF-*β*1 treated (dark gray bar), and TGF-*β*1 + SB525334 treated cells (light gray bar), ***P ≤ 0.001, **P ≤ 0.005, or *P ≤ 0.05. Scale bars = 100 *μ*m.

TGF-*β*1 and TGF-*β*R1 stainings highlighted acinar formation in all three groups (Figures 8(a), 8(c), 8(d)–8(f), 8(g), 8(i), 8(j)–8(l)). In matrigel and TGF-*β*1 + SB525334, acini-like structures were formed in both areas rich in mesenchymal cells (Figures 8(a), 8(g) and 8(c), 8(i)) and also mesenchymal free area (Figures 8(d), 8(j) and 8(f), 8(l)) whereas acini-like structures in TGF-*β*1 group were not found in the areas rich in mesenchymal cells (Figures 8(b) and 8(h)). Larger acini-like structures with ductal-like structures were found in TGF-*β*1 + SB525334 (Figures 8(f), 8(l) arrowheads) compared to those in the TGF-*β*1 (Figures 8(e) and 8(k)) and control (matrigel only) groups (Figures 8(d) and 8(j)).

 The expression of *Tgf-b1* and *Tgf-br1* was also analyzed after *in vitro* differentiation and compared between the three different groups (Figures 8(m) and 8(n)). TGF-*β*1 induced up-regulation of both *Tgf-b1* and *Tgf-br1* expression whereas inhibition of TGF-*β*1 signaling in the TGF-*β*1 + SB525334 resulted in significant downregulation of both *Tgf-b1* and *Tgf-br1 *at day 3 and whereas at day 5, *Tgf-b1* was still downregulated both not its receptor, *Tgf-br1*.

## 4. Discussion

Although the epithelium of the parotid glands is ectoderm-derived whereas the epithelium of the submandibular and sublingual glands is endoderm-derived, the salivary gland mesenchyme is neural crest-derived [43]. The interactions of epithelium and mesenchyme are essential for the branching morphogenesis of the salivary gland. Molecular cues such as secretion of fibroblast growth factors (FGF-10, FGF-7) by the ectomesenchyme and expression of FGF receptors (FGF-R1, FGF-R2) by the epithelium are important for the development salivary gland [5, 43–48]. Also other morphogens such as Shh and Wnt are also important for the saliva gland development [49–51]. Although the role of the mesenchyme during salivary gland development is well studied, the role of mesenchyme in adult salivary gland tissue homeostasis and its potential involvement in salivary diseases is understudied. 

Therefore, we sought to better characterize the phenotype of salivary gland mesenchyme and to develop a culture system to *in vitro* study the interactions of adult salivary gland epithelial cells and mesenchymal cells. We used the transgenic *Col1a1-GFP* reporter mice to identify mesenchymal cells in the salivary gland [26]. Upon histological analysis of the *Col1a1-GFP* mice we can exclusively identify mesenchymal cells as GFP-positive cells in salivary glands.

*Ex vivo* cultures of the mixed salivary gland cells in N2 versus DMEM plus serum media revealed the presence of both epithelial and mesenchymal (GFP-positive) cells in culture. Cells in N2 medium stopped proliferating early in culture whereas the cells in DMEM plus serum medium continued proliferating for more than 10 passages. The levels of TGF-*β*1 expression in early cultures are higher in N2 medium which may explain why cells cultured in this medium stopped proliferating early in culture. RT-PCR and immunohistochemical analysis of the DMEM plus serum cultures at several passages revealed the presence of both epithelial and mesenchymal cells even in late cultures. Upon differentiation on matrigel the mixed culture cells were able to differentiate into mature acini-like structures. 

Therefore, this culture condition allows the long-term expansion of both epithelial and mesenchymal cells which in turn can be induced to differentiate *in vitro*. This culture system offers many advantages over existing culture conditions: (1) long-term expansion of mesenchymal cells, (2) long-term expansion of epithelial cells, and (3) *in vitro* differentiation of mixed cultured cells to study epithelial-mesenchymal interactions and effects of inducers and inhibitors. 

The effects of TGF-*β*1 on the salivary glands are complex and somewhat paradoxical. TGF-*β* null mice developed multifocal infiltrates in heart, lungs and salivary glands [18, 52]. These multifocal lesions and resulting damage to salivary glands were gender-specific and not only caused by defects in T-cell suppression but also by defects in TGF-*β* signaling in salivary gland epithelial cells, as in another study it was shown that conditional deletion of TGF-*β*R1 on salivary gland epithelial cells using the mammary tumor virus *Cre* mouse led to salivary gland inflammatory lesions and abnormal pattern of aquaporin-5 distribution, resulting in saliva secretion defects only in females but not in males mice [53]. On the other hand, conditional over-expression of TGF-*β*1 in secretory cells (mammary and salivary gland epithelial cells) resulted in hyposalivation due to salivary gland fibrosis and atrophy [21]. We hypothesized these paradoxical effects may be explained by differential roles of TGF-*β* signaling in different salivary gland cell types, namely epithelial cells versus mesenchymal cells. Therefore, we tested the utility of our mixed cell culture system to study the effects TGF-*β*1 *in vitro*. 

Supplementation of TGF-*β*1 to mixed cell cultures induced expression of higher levels of acini markers; however, we also observed reduction of mesenchymal derived fibroblast growth factors (*Fgf-7* and *Fgf-10*) and dramatic increased in the procollagen type 1 levels. FGF-7 (aka, Keratinocyte Growth Factor, KGF) in particular has been proven beneficial for salivary gland epithelial differentiation. *In vitro* salivary gland explants in the absence of mesenchyme can undergo differentiation with EGF and FGF-7. EGF induced lobule formation whereas FGF-7 induced stalk elongation morphogenesis [54]. FGF-7 has been shown beneficial for salivary gland restoration [55, 56]. Transgenic mice expressing *Fgf-7* under the keratin (K14) promoter exhibited excessive salivation [57]. 

Histological examination of the differentiation cultures revealed lack of acini-like structures formation and undifferentiated appearance of epithelial cells adjacent to mesenchymal cells which in turn adapted a fibroblasts morphology (elongated and polarized) in the TGF-*β*1 induced group. In contrast, inhibition of TGF-*β*R1 signaling with SB525334 results in the highest levels of acini markers, highest levels of *Fgfs*, and lowest levels of *collagen type I*, which corresponds with the most mature and largest acini-like structures, especially in the areas rich in mesenchymal cells which adapted a cobblestone morphology. Thus we conclude that inhibition of TGF-*β*1 signaling in these *in vitro* differentiation cultures is beneficial, particularly in mesenchymal cells. 

 Alterations in the TGF-*β* signaling have been associated with several salivary gland disorders. Sjogren's syndrome is an autoimmune disorder characterized primarily by T-cell but also B-cell infiltration in the salivary glands. Immunohistochemical staining showed TGF-*β*1 was strongly expressed in ductal epithelial cells of normal and inflamed salivary glands but downregulated in Sjogren's salivary glands [22]. In another study, normal and Sjogren's salivary glands expressed TGF-*β* in ductal and acinar epithelial cells, but TGF-*β* production was reduced in Sjogren's salivary gland cultures [58]. Another study showed all three isoforms of TGF-*β* expressed in lymphocytes, endothelial cells and ductal cells of Sjogren's versus benign lymphoepithelial lesions. Interestingly, the expression of TGF-*β* isoforms in ductal cells of Sjogren's was increased as compared to benign lesions [59]. These reports suggest that alterations of TGF-*β*1 pathway may be involved in pathogenesis of Sjogren's disease

Alterations of the TGF-*β* pathway and abnormal expression of TGF-*β* ligands and receptors have been reported in several salivary gland tumors. In pleomorphic adenomas (PA), the most common type of salivary gland tumors, TGF-*β*2 was expressed in the inner ductal cells and TGF-*β*3 was expressed in the myoepithelial cells of PA tumors [60]. Mucoepidermoid carcinoma (MEC) is another salivary gland tumor that exhibits differentiation in multiple lineages. TGF-*β*1 was expressed in the salivary gland ducts, stroma and endothelial cells of the MEC tumors. Interestingly, TGF-*β*R2 expression inversely correlated with tumor grade: all low grade tumors showed expression of TGF-*β*R2 whereas none of the high-grade tumors, with greatest metastatic potential, showed TGF-*β*R2 expression. TGF-*β*R2 was expressed in surface epithelium, endothelial cells, nonneoplastic salivary gland ducts and stromal fibroblasts of the low-grade MEC [61]. Loss of TGF-*β*R2 expression correlated with loss of tumor differentiation. In another study, it was shown that the Ms cell line derived from MEC, which highly expressed TGF-*β*1, exhibited decreased invasion and migration capacity when TGF-*β*1 was silenced by siRNA [24]. Likewise, another study showed that TGF-*β*1 was highly expressed in a metastatic salivary adenoid cystic carcinoma cell line and exposure to TGF-*β*1 *in vitro* activated the classical TGF-*β* pathway, suggesting that TGF-*β*1 may promote migration and invasion of this tumor [19]. This suggests that the expression of TGF-*β*1 on certain tumor salivary gland epithelial cells induces migration, invasion and metastasis, and that TGF-*β*1 inhibition in these tumors may be beneficial. 

In summary, given the complexity of TGF-*β* signaling in salivary gland development, homeostasis and diseases, better tools are needed to understand the differential effects and role of TGF-*β* signaling in different salivary gland cells (epithelial versus mesenchymal cells). The emergence of tissue specific conditional knockouts such as* Cre-lox *recombination [62], specific for salivary gland mesenchyme versus epithelium are necessary to dissect apart the differential roles of TGF-*β* signaling. 

## Figures and Tables

**Table 1:** Mouse-specific primer sequences.

Gene	Forward primer (5′→3′)	Reverse primer (5′→3′)	GenBank accession number
*Gapdh *(R)	CTCGTCCCGTAGACAAAATGG	CGCTCCTGGAAGATGGTG	NM_008084
*Gapdh *(Q)	GGGAAGCCCATCACCATCT	GCCTCACCCCATTTGATGTT	NM_008084
*Amylase-1(Amy-1) *(B)	GGTGCAACAATGTTGGTGTC	ACTGCTTTGTCCAGCTTGAG	NM_007446
*Aqp-5 *(B)	CGACCGTGTGGCTGTGGTCA	GTGCCGGTCAGTGTGCCGTC	NM_009701
*Collagen type I *(B)	ACGGCTGCACGAGTCACAC	GGCAGGCGGGAGGTCTT	NM_007742
*Fgf-7 *(R)	ACTGTTCCAGCCCCGAGCGA	TTCCCCTCCGCTGTGTGTCCA	NM_008008
*Fgf-7 *(Q)	GTCCGGAGCAAACGGCTACGA	TGTGTCGCTCGGGGCTGGAA	NM_008008
*Fgf-10* (R)	CGCAGAGGGGCGCAGATGTC	GCCTGTCCTCGCTCCGTCCT	NM_008002
*Fgf-10* (Q)	TGGTGTCACAGGAGGCCACCAA	CGCACATGCCTTCCCGCACT	NM_008002
*Pdgfr-a* (R)	TTTGTGCCTCTCGGGATGA	TGACGGGCAGCACATTCA	NM_011058
*Occludin *(Q)	AGACCCAAGAGCAGCCAAAG	GGAAGCGATGAAGCAGAAGG	NM008756
*Tgf-b1 *(Q)	CTACTATGCTAAAGAGGTCACC	TTTCTCATAGATGGCGTTGTTGC	NM_011577
*Tgf-br1 *(Q)	GGAAATTGCTCGACGCTGTT	TTCTCATTTCTTCAACCGATGGA	NM_009370
*ZO-1 *(Q)	CGAGGCATCATCCCAAATAAGAAC	TCCAGAAGTCTGCCCGATCAC	NM_009386

Note: (R): primer sequences used for RT-PCR; (Q): primer sequences used for Q-RT-PCR; (B): primer sequences used for both RT-PCR and Q-RT-PCR.

**Table 2:** Antibody used for immunohistochemical staining.

Marker	Antibody	Species	Dilution	Company
AMY-1	Polyclonal	Rabbit	1 : 50	Thermo Scientific
AQP-5	Polyclonal	Rabbit	1 : 50	Calbiochem
CD44-PE	Monoclonal	Rat	1 : 400	eBioscience
Collagen type I	Polyclonal	Rabbit	1 : 400	Abcam
E-cad-biotin	Monoclonal	Rat	1 : 400	eBioscience
LAMP-1	Monoclonal	Rat	1 : 100	Developmental Hybridoma Bank
S100	Polyclonal	Rabbit	1 : 400	Dako
SMA-Cy3	Monoclonal	Mouse	1 : 400	Sigma
TGF-*β*1	Polyclonal	Rabbit	1 : 100	Abcam
TGF-*β*R1	Polyclonal	Rabbit	1 : 100	Millipore
TGF-*β*R2	Polyclonal	Rabbit	1 : 50	Millipore
vWF	Polyclonal	Rabbit	1 : 100	Dako
